# Confidence limits for patient‐specific IMRT dose QA: a multi‐institutional study in Korea

**DOI:** 10.1120/jacmp.v17i1.5607

**Published:** 2016-01-08

**Authors:** Jung‐in Kim, Jin‐Beom Chung, Ju‐Young Song, Sung Kyu Kim, Yunseok Choi, Chang Heon Choi, Won Hoon Choi, Byungchul Cho, Jin Sung Kim, Sung Jin Kim, Sung‐Joon Ye

**Affiliations:** ^1^ Department of Radiation Oncology Seoul National University Hospital Seoul; ^2^ Department of Radiation Oncology Seoul National University Bundang Hospital Seongnam; ^3^ Department of Radiation Oncology Chonnam National University Medical School Hwasun; ^4^ Department of Therapeutic Radiology & Oncology Yeungnam University Seoul; ^5^ Department of Radiation Oncology Jeju National University Hospital Jeju; ^6^ Department of Radiation Oncology Veterans Health & Service Medical Center Seoul; ^7^ Department of Radiation Oncology Yonsei Cancer Center, Yonsei University College of Medicine, Yonsei University Health System Seoul; ^8^ Department of Radiation Oncology Asan Medical Center, University of Ulsan College of Medicine Seoul; ^9^ Department of Radiation Oncology Seoul Samsung Medical Center Seoul; ^10^ Department of Radiation Oncology Eulji University Hospital Daejon; ^11^ Department of Transdisciplinary Studies and Advanced Institutes of Convergence Technology Seoul National University Seoul Korea

**Keywords:** confidence limit, IMRT dose QA, correlation, multi‐institutional study

## Abstract

This study aims to investigate tolerance levels for patient‐specific IMRT dose QA (DQA) using the confidence limits (CL) determined by a multi‐institutional study. Eleven institutions participated in the multi‐institutional study in Korea. A total of 155 DQA measurements, consisting of point‐dose differences (high‐ and low‐dose regions) and gamma passing rates (composite and per‐field) for IMRT patients with brain, head and neck (H&N), abdomen, and prostate cancers were examined. The Shapiro‐Wilk test was used to evaluate the normality of data grouped by the treatment sites and the DQA methods. The confidence limit coefficients in cases of the normal distribution, and the two‐sided Student's *t*‐distribution were applied to determine the confidence limits for the grouped data. The Spearman's test was applied to assess the sensitivity of DQA results within the limited groups. The differences in CLs between the two confidence coefficients based on the normal and *t*‐distributions were negligible for the point‐dose data and the gamma passing rates with 3%/3 criteria. However, with 2%/2 criteria, the difference in CLs were 1.6% and 2.2% for composite and per‐field measurements, respectively. This resulted from the large standard deviation and the more sensitive criteria of 2%/2. There was no noticeable correlation among the different QA methods. Our multi‐institutional study suggested that the CL was not a suitable metric for defining the tolerance level when the statistics of the sample group did not follow the normality and had a large standard deviation.

PACS number: 87.55.Qr

## I. INTRODUCTION

The American Association of Physicists in Medicine (AAPM) commissioned the Task Group (TG) 119 multi‐institutional study for the purpose of evaluating intensity‐modulated radiation therapy (IMRT) commissioning.^(1)^ TG‐119 proposed the concept of the confidence limit (CL) assuming a normal distribution of collected data, and suggested the tolerance level of point and planar dose measurements for their mock structures. However, the results of planar dose quality assurance (DQA) showed a large standard deviation, resulting in a low tolerance level. Further, unlike percent differences in point‐dose QA, the gamma passing rate for planar dose QA has ideal lower and upper bounds of 0% and 100%, respectively. In this case, the upper bound prevented the measured data from following a normal distribution. Thus, Knill and Snyder^(2)^ studied the limitations of the confidence limit as suggested in the TG‐119 report when applying the gamma passing rate. They reported that differences in the confidence limits based on the truncated normal distribution and the Weibull distribution as asymmetric fitting were within 0.1%. The Knill study collected data from a sole local institution. However, consensus values for tolerance or action levels should be derived from a multi‐institutional study. Thus multiple institutions have been involved in this study to suggest appropriate tolerance levels for IMRT DQA and planning.^1^, ^3^, ^4^, ^5^, ^6^, ^7^ In statistics, when a sampling distribution is approximately normal, the range of values between $\bar{x}\pm 1.96\sigma$ is called the 95% confidence interval for a sample where $\bar{x},\;1.96$, and σ are a mean value, confidence coefficient, and standard deviation, respectively. The two boundaries of the interval of $\bar{x}‐1.96\sigma$ and $\bar{x}+1.96\sigma$ are called the 95% confidence limits. Due to a small sample size, when accurate determination of the standard deviation of the population may not be possible, the confidence coefficients found using the Student's *t*‐distribution can be applied.^8^, ^9^ The methods widely adopted in multi‐institutional studies of IMRT DQA are point‐dose measurements and gamma evaluation of 2D dose distribution.^1^, ^3^, ^4^, ^5^, ^10^ Nelms et al.^(11)^ showed that planar gamma passing rates did not predict clinically relevant patient dose errors in anatomic regions‐of‐interest. Stasi et al.^(12)^ also studied a correlation between gamma passing rates and DVH information in patient‐specific IMRT DQA. They concluded that there was no such correlation between them. However, none of these studies used a multi‐institutional methodology to address the correlations among different DQA methods.

A national multi‐institutional study to address these concerns was organized into a series of two programs. The first, called a mock program, was to evaluate the commissioning status of each institution using the AAPM TG‐119 methodology.^(4)^ Next was a clinical program, which used clinical cases from the qualified institutions. The current study was based on the results of the clinical program. The normality of the sample data was tested and the correlation between the different QA methods was statistically addressed. The determined CL was used to derive the tolerance levels.

## II. MATERIALS AND METHODS

### A. Multi‐institutional study

This multi‐institutional study was performed from October 2011 to September 2012 and comprised of data from 11 institutions in Korea. These institutions had been strictly evaluated for the commissioning status of treatment planning system (TPS) and the adequacy of QA systems in the previous multi‐institutional mock program.^(4)^ This study was composed with clinical IMRT cases of brain, H&N, abdomen, and prostate treatments from each institution. These data were routine QA results of the patients and have been taken randomly for analysis. The total 155 cases included 20, 60, 18, and 57 brain, H&N, abdomen, and prostate plans, respectively. DQA performance consisted of a point‐dose and a planar dose measurement, using locally available equipment. Table 1 summarizes the TPS, delivery systems, and QA equipment for planar dose measurements used at each institution.

**Table 1:** List of participating institutions, TPS, delivery systems, and QA equipment for planar dose measurements.

*Institution*	*Accelerator*	*TPS*	*Delivery Technique*	*QA Equipment*
Seoul Nat'l Univ. Hosp.	Varian IX	Eclipse 8.9	Dynamic MLC	2DArray
Seoul Nat'l Univ. Bundang Hosp.	Varian 21ExS	Eclipse 6.5	Dynamic MLC	MapCHECK
Jeju Nat'l Univ. Hosp.	Varian IX	Eclipse 8.6	Dynamic MLC	MapCHECK2
Yeungnam Univ. Hosp.	Varian 21ExS	Eclipse 8.6	Dynamic MLC	MatriXX
Kangbuk Samsung Medical Center	Varian IX	Eclipse 8.9	Dynamic MLC	MatriXX
Eulji Univ. Hosp.	Elekta Synergy	Monaco 2.0.3	Static MLC	ArcCHECK
Veterans Health & Service Medical Center	Varian IX	Eclipse 8.9	Dynamic MLC	MatriXX
Asan Medical Center	Varian Trilogy	Eclipse 8.9	Dynamic MLC	MatriXX
Seoul Samsung Medical Center	Tomotherapy	TPS 3.1.4	Binary MLC	EBT2
Yonsei Cancer Center	Tomotherapy	TPS 4.0.2	Binary MLC	EBT2
Chonnam Nat'l Univ. Hwasun Hosp.	Tomotherapy	TPS 3.2.3.2	Binary MLC	EBT2

### B. Dose quality assurance

The point doses were measured in both the planning target volume (PTV) and the normal organ. The point dose to PTV was approximately 95%∼105% of the prescription dose and measured as a high dose. The point dose to the normal organ was typically 30%∼50% of the prescription dose with low gradient region and measured as a low dose. The dose difference value (%) was expressed as a ratio of the difference between measured and calculated doses to the calculated dose. The institutional measurements performed on linacs used a 0.125 cc ion chamber (Semiflex, PTW, Freiburg, Germany) and a custom‐made phantom of acrylic developed in our previous mock program to minimize the equipment dependency. The institutional measurements performed using tomotherapy utilized a 0.05 cc ion chamber (Exradin A1SL, Standard Imaging Inc., Middleton, WI) and the commercial phantom (i.e., ‘cheese’ phantom, Accuracy, Sunnyvale, CA).

The planar dose distributions were measured in the composite and per‐field irradiations. The per‐field measurements were done only with the linac‐based institutions. Each institution used either a detector array or film, as summarized in Table 1. Two institutions used the MapCHECK device (Sun Nuclear Corporation, Melbourne, FL), one institution used an ion chamber array (PTW, Freiburg, Germany), four institutions used MatriXX (IBA Dosimetry GmbH, Schwarzenbruck, Germany), one institution used ArcCHECK (Sun Nuclear), and three institutions used EBT2 films (International Specialty Products, Wayne, NJ). Each institution was provided with all performance information about the planar dose QA from calibration to measurement. The gamma index was evaluated with two separate criteria, 2%/2 and 3%/3, with the global criteria using available software of each institution. The gamma comparison was performed with a low‐dose threshold such that any pixels that received less than 10% of the maximum dose in the dose map were excluded in the evaluation.^(13)^ The excluded points were outside of the region of interest (ROI).

### C. Statistical analysis

The results of multi‐institutional IMRT QA were grouped depending on the QA methodologies for each clinical case. For each group, the normality was evaluated with the Shapiro‐Wilk test to determine whether the dataset is well‐modeled by a normal distribution.^(14)^ The value of the CLs was calculated with the mean, confidence coefficient, and standard deviation values. Two confidence coefficients (i.e., 1.96 and two tails of *t*‐distributions) were selected for calculating and comparing the CLs for each group. The confidence coefficient based on the *t*‐distribution was determined by the degrees of freedom as n‐1 (n referring to the number of samples). However, the confidence coefficient of 1.96 was effective only for the groups shown to follow the normal distribution.

The sensitivity of the confidence coefficient was analyzed by comparing the values of the CLs. The Spearman's correlation coefficient was used to evaluate the correlation among the high‐dose point, low‐dose point, and composite field with 3%/3 criteria.

## III. RESULTS

### A. Point‐dose QA and confidence limit

For the high‐dose measurements, the data grouped by H&N and abdomen followed the normal distribution, as confirmed by the Shapiro‐Wilk test (p>0.05). The averaged values of dose differences for brain, H&N, abdomen, and prostate groups were −0.1%±1.3%, 0.0%±1.5%, −0.4%±1.4% , and −0.2%±1.1%, respectively. The corresponding CLs were from 2.3% to 3.1%, with normal confidence coefficient from 2.4% to 3.3% based on the *t*‐distribution. The maximum difference in CLs was 0.2% in the abdomen group. The Student's *t*‐distribution was more appropriate to groups with small numbers of samples, generally less than 30. The average dose difference over all cases was −0.1%±1.3% and the corresponding CL was 2.7%. For the low‐dose measurements, the data grouped by brain and abdomen followed the normal distribution, as confirmed by the Shapiro‐Wilk test (p>0.05). The averaged values of dose differences for brain, H&N, abdomen, and prostate groups were −1.9%±3.4%,−0.8%±2.1%,−0.8%±3.0%, and −1.4%±2.5%, respectively. The corresponding CLs were from 5.0% to 8.5%, with normal confidence coefficients from 5.0% to 9.0% based on the *t*‐distribution. The maximum difference in CLs was 0.5% in the brain and abdomen groups. The average dose difference over all cases was −1.1%±6.2%, which showed a larger deviation than did the high‐dose measurement, and the corresponding CL was 6.2%. The results of point‐dose measurements are summarized in Table 2.

**Table 2:** The summary of point‐dose measurement and analysis.

*Cases*		*Brain*	*H&N*	*Abdomen*	*Prostate*	*Total*
n		20	60	18	57	155
Normal Coefficient^a^		1.96	1.96	1.96	1.96	1.96
Two‐tail Coefficient^b^		2.093	2.001	2.110	2.003	1.975
	Shapiro‐Wilk	0.025	0.089	0.825	0.001	0.001
	Mean	‐0.1%	0.0%	‐0.4%	‐0.2%	‐0.1%
High dose	SD	1.3%	1.5%	1.4%	1.1%	1.3%
	CL^a^	2.7%	3.0%	3.1%	2.3%	2.7%
	CL^b^	2.8%	3.0%	3.3%	2.4%	2.7%
	Shapiro‐Wilk	0.286	0.002	0.082	0.001	0.000
	Mean	‐1.9%	‐0.8%	‐0.8%	‐1.4%	‐1.1%
Low dose	SD	3.4%	2.1%	3.0%	2.5%	2.6%
	CL^a^	8.5%	5.0%	6.6%	6.3%	6.2%
	CL^b^	9.0%	5.0%	7.1%	6.4%	6.2%

^a^ Confidence limit based on normal distribution.

^b^ Confidence limit based on *t*‐distribution.

H&N = head and neck; SD = standard deviation.

### B. Planar dose QA and confidence limit

Table 3 presents a summary of the results of the composite field measurement and analysis. None of the groups followed the normal distribution per the Shapiro‐Wilk test (p<0.05) with both criteria of gamma analysis. However, the confidence coefficient of 1.96 was also applied to compare the CLs between different fitting curves. The averaged gamma passing rates with 2%/2 mm criteria for brain, H&N, abdomen, and prostate groups were 92.4%±7.7%,90.9%±8.1%,89.5%±10.7%, and 92.6%±5.4%, respectively. The corresponding CLs were from 68.5% to 82.1% with the normal confidence coefficient and from 66.9% to 81.8% based on the *t*‐distribution. The maximum difference in CLs was 1.6% in the abdomen group. The average gamma passing rate over all cases was 91.6%±7.5% and the corresponding CL was 76.9%. With 3%/3 mm criteria, the averaged gamma passing rates for brain, H&N, abdomen, and prostate groups were 98.3%±2.4%, 97.8%±2.2%, 98.0%±2.2%, and 97.6%±2.0%, respectively. The corresponding CLs were from 93.5% to 93.7% with the normal confidence coefficient and from 93.3% to 93.5% based on the *t*‐distribution. The maximum difference in CLs was 0.4% in brain group. The average gamma passing rate over all cases was 97.8%±2.1% and the corresponding CL was 93.6%.

Table 4 shows a summary of the per‐field measurement and analysis. Per‐field measurements were performed only for the linac‐based group. Only the group for brain followed the normal distribution per the Shapiro‐Wilk test (p>0.05) with two different criteria analysis. However, the confidence coefficient of 1.96 was also applied to compare the CLs between different fitting curves. The averaged gamma passing rates with 2%/2 mm criteria for brain, H&N, abdomen, and prostate groups were 93.9%±6.0%, 91.3%±4.2%, 90.7%±7.3%, and 92.9%±3.9%, respectively. The corresponding CLs were from 76.5% to 85.1% with the normal confidence coefficient and from 74.3% to 84.9% based on the *t*‐distribution. The maximum difference in CLs was 2.2% in the abdomen group. The average gamma passing rate over all cases was 97.5%±4.8% and the corresponding CL was 82.8%. With 3%/3 mm criteria, the averaged gamma passing rates for brain, H&N, abdomen, and prostate groups were 98.1%±2.7%, 97.3%±1.9%, 97.7%±1.9%, and 97.5%±1.7%, respectively. The corresponding CLs were from 92.9% to 94.2% with the normal confidence coefficient and from 92.4% to 94.1% based on the *t*‐distribution. The maximum difference in CLs was 0.6% in the brain group. The average gamma passing rate over all cases was 97.5%±1.9% and the corresponding CL was 93.7%.

**Table 3:** The summary of composite field measurement and analysis.

*Cases*		*Brain*	*H&N*	*Abdomen*	*Prostate*	*Total*
n		20	60	18	57	155
Normal Coefficient^a^		1.96	1.96	1.96	1.96	1.96
Two‐tail Coefficient^b^		2.093	2.001	2.110	2.003	1.975
	Shapiro‐Wilk	0.005	0.000	0.004	0.000	0.000
	Mean	92.4%	90.9%	89.5%	92.6%	91.6%
2%/2 mm	SD	7.7%	8.1%	10.7%	5.4%	7.5%
	CL^a^	77.3%	75.0%	68.5%	82.1%	76.9%
	CL^b^	76.2%	74.6%	66.9%	81.8%	76.8%
	Shapiro‐Wilk	0.000	0.000	0.016	0.001	0.000
	Mean	98.3%	97.8%	98.0%	97.6%	97.8%
3%/3 mm	SD	2.4%	2.2%	2.2%	2.0%	2.1%
	CL^a^	93.7%	93.5%	93.7%	93.6%	93.6%
	CL^b^	93.3%	93.4%	93.4%	93.5%	93.6%

^a^ Confidence limit based on normal distribution.

^b^ Confidence limit based on *t*‐distribution.

H&N = head and neck; SD = standard deviation.

**Table 4:** The summary of per‐field measurement and analysis.

*Cases*		*Brain*	*H&N*	*Abdomen*	*Prostate*	*Total*
n		14	22	10	45	91
Normal Coefficient^a^		1.96	1.96	1.96	1.96	1.96
Two‐tail Coefficient^b^		2.160	2.080	2.262	2.015	1.987
	Shapiro‐Wilk	0.011	0.226	0.089	0.201	0.001
	Mean	93.9%	91.3%	90.7%	92.9%	97.5%
2%/2 mm	SD	6.0%	4.2%	7.3%	3.9%	4.8%
	CL^a^	82.1%	83.1%	76.5%	85.1%	83.0%
	CL^b^	80.9%	82.6%	74.3%	84.9%	82.8%
	Shapiro‐Wilk	0.000	0.124	0.130	0.126	0.000
	Mean	98.1%	97.3%	97.7%	97.5%	97.5%
3%/3 mm	SD	2.7%	1.9%	1.9%	1.7%	1.9%
	CL^a^	92.9%	93.6%	93.9%	94.2%	93.8%
	CL^b^	92.4%	93.3%	93.3%	94.1%	93.7%

^a^ Confidence limit based on normal distribution.

^b^ Confidence limit based on t‐distribution.

H&N = head and neck; SD = standard deviation.

### C. Correlation of QA results

The statistical correlation among the results of different DQA methods was assessed using the Spearman's correlation coefficient and presented as *r*‐values. The coefficients are presented in Table 5, together with relevant *p*‐values. The *r*‐values of high‐dose point versus low‐dose point and composite field evaluation were 0.106 (p=0.187) and 0.016 (p=0.84), respectively. The *r*‐value of low‐dose point versus composite field evaluation was 0.04 (p=0.618). The absolute *r*‐values were always less than 0.8, which often indicated no strong correlation between them.

**Table 5:** Spearman's correlation coefficient (*r*‐value) among different dose QA methods (criteria of 3%/3 mm for composite field measurement).

*Measurement*		*Low Dose*	*Composite Field*
High dose	r‐value	0.106	0.016
	p‐value	0.187	0.840
	r‐value		0.04
Low dose	p‐value		0.618
	n	155	155

## IV. DISCUSSION

AAPM TG‐119 adopted a concept of CLs with the assumption that IMRT DQA data follows a normal distribution, and suggested tolerance levels of point and planar dose measurements as a guide for IMRT commissioning. Instead of the mock structures, we collected patient‐specific DQA data from multiple institutions and grouped the data by the treatment sites and DQA methods. In our previous mock study,^(4)^ DQA results in point and planar dose measurements followed the normal distribution, per the Shapiro‐Wilk test (p>0.05). In this study, however, the groups following the normal distribution per the Shapiro‐Wilk test (p>0.05) were the high‐dose point DQA for H&N and abdomen sites and the low‐dose point DQA for brain and abdomen sites. The groups following the normal distribution per the Shapiro‐Wilk test (p>0.05) were the per‐field DQA for the H&N, abdomen, and prostate sites. Most of the measured groups did not follow the normal distribution. The difference in group normality tests between the clinical and mock programs resulted from the fact that identical structures were used in the mock program, whereas the clinical program was based on diverse patient‐specific anatomy. The tolerance levels of point DQA were comparable for the two programs, as the results of both programs followed the normal distribution and showed similar standard deviations. In this study, the result in low‐dose regions had a larger standard deviation than that in high dose regions since the low dose regions in general were not uniform and had more uncertainty of dose calculation than the high‐dose regions. For the planar DQA, the tolerance levels were higher in the clinical program by 4% to 5% than those in our previous mock program, even though the grouped results of the clinical program did not follow the normal distribution. In general, the per‐field measurement has a larger uncertainty than the composite field measurement. The CL was calculated with the average value and standard deviation. In this study, however, the number of samples for the per‐field measurement was much larger than that of the composite field measurement. Thus, the per‐field measurement showed the outcome with a smaller standard deviation than the composite measurement.

In this study, the CL is also taken as a tolerance level. The CL is a function of the standard deviation of a normal distribution. As noted in the TG‐119 report, the data of gamma‐index passing rate may be invalid for establishing CLs because of the assumption that measured data follow a normal distribution. Thus we tested the normality of the measured data for each DQA method in advance and then adopted the appropriate distribution for a confidence coefficient. However, the difference in CLs, calculated using the TG‐119 approximation and *t*‐distribution, was not noticeable (>2%). Knill and Snyder^(2)^ also reported the maximum expected difference in CLs was 1.2% with a truncated distribution. The concept of the confidence limit was not a perfect metric in determining the tolerance level because it was strongly dependent on the standard deviation and whether data follow the normality or not. Nevertheless, in this clinical program, the difference in CLs between the two confidence coefficients based on the normal and *t*‐distributions was negligible in the point‐dose analysis and the planar dose gamma evaluation with 3%/3 mm criteria, which were 0.2% and 0.5%, 0.4%, and 0.6% for high dose, low dose, composite field, and per‐field measurements, respectively. However, with 2%/2 mm criteria, the difference in CLs were 1.6% and 2.2% for composite and per‐field measurements, respectively. This resulted from the large standard deviation and more sensitive criteria of 2%/2 mm. Pulliam et al.^(15)^ reviewed the huge DQA results of more than 13,000 from 13 different sites using 90% passing 5%/3 mm global agreement criteria. They said that the high threshold of 3%/3 mm, which is commonly used with planar QA, was not clear for evaluating whether the plans acceptable or not. Stasi et al.^(12)^ demonstrated that the results of various QA methods were not strongly correlated with one another and asserted that the published acceptance criteria have a disputable predictive power for patient‐specific IMRT QA. Similarly, in this study there was no correlation among different DQA methods. This implies that the result from one QA method may not predict the results of other QA methods. On the other hand, there have been many other approaches to develop the metrics to predict the deliverability of plans and DQA results. They included the studies on modulation indices,^16^, ^17^, ^18^ texture analysis,^19^, ^20^ machine‐learning features,^21^, ^22^ all of which have been under development.

The goal of DQA is to detect unacceptable plans that may contain a large deviation between planned and delivered doses. To date, none of devices and methodologies is the standard for DQA and, further, the related communities have not reached the common consensus on it. In this respect, this current work based on a multi‐institutional study provides at least a judge on the suitability of CL as tolerance levels for IMRT DQA.

## V. CONCLUSIONS

Our multi‐institutional study suggested that the CL was not a suitable metric for defining the tolerance level when the statistics of the sample group did not follow the normal distribution and had a large standard deviation. However, the results of this study can be used as a comparison guide for other institutions to evaluate their IMRT DQA results.

## ACKNOWLEDGMENTS

This work was in part supported by the National Research Foundation of Korea (490‐20150036, 490‐20140041 and 5267‐20150100) grant funded by the Korea government. The authors are grateful to the editor and associate editors for their valuable comments and review of this paper.
